# Retrospective Study of Japanese Patients with Schizophrenia Treated with Aripiprazole

**DOI:** 10.5402/2012/454898

**Published:** 2012-08-30

**Authors:** Tetsuya Tanioka, Syoko Fuji, Mika Kataoka, Beth King, Masahito Tomotake, Yuko Yasuhara, Rozzano Locsin, Keiko Sekido, Kazushi Mifune

**Affiliations:** ^1^Department of Nursing, Institute of Health Biosciences, The University of Tokushima Graduate School, Tokushima 770-8509, Japan; ^2^Christine E. Lynn College of Nursing, Florida Atlantic University, Boca Raton, FL 33431, USA; ^3^Department of Psychiatry, Mifune Hospital, Kagawa 763-0073, Japan

## Abstract

*Aim*. The purpose of this retrospective study was to evaluate changes in clinical indicators which influence the quality of life (QOL) of patients with schizophrenia treated by antipsychotic therapy before and after switching to aripiprazole. *Methods*. A retrospective chart review of 27 patients diagnosed with schizophrenia and who were switched from one antipsychotic to aripiprazole was performed. Clinical indicators about the daily dosage of antipsychotics and antiparkinsonian drugs, psychiatric condition, and glucose/lipid metabolism, clinical evaluation by nursing observation were used to measure the responsiveness of subjects to aripiprazole. *Results*. Of the 27 subjects, 14 responded to the switch to aripiprazole with significant improvement of the Brief Psychiatric Rating Scale (BPRS) score (*P* = 0.04), significant decrease in dosage of antipsychotics in 71% of patients (*P* = 0.03), and tendency toward reduction in dosage of antiparkinsonian drugs (*P* = 0.07) and body mass index (BMI) (*P* = 0.06). However, 8 of 27 subjects had a significant increase in lipid levels after switching to aripiprazole (*P* = 0.01). *Conclusion*. QOL for subjects who responded to the switch to aripiprazole improved as indicated by lower doses of antipsychotic and antiparkinson medications, improvement in BPRS score, and a decrease in BMI. Results indicate little influence on patient's QOL.

## 1. Introduction

The development of new antipsychotic medications provided the opportunity to enhance the quality of life (QOL) of persons living with schizophrenia, a chronic, disabling brain disorder. The National Institute of Mental Health reports that about 1% of adults living in the United States have schizophrenia [1]. According to Japan's Ministry of Health, Labor and Welfare, this statistic is similar to Japan's which is around 0.7% [2]. Aripiprazole, an atypical antipsychotic, has been reported to enhance the quality of life for those persons living with schizophrenia. 

In clinical studies, atypical antipsychotics are associated with greater improvements in quality of life and subjective well-being than are typical antipsychotics [3]. When measuring QOL in patients treated with antipsychotics, it is important to acknowledge that a variety of factors may influence a patient's QOL: these include side effects and daily dosage of the antipsychotic, depressive, and negative symptoms, duration of treatment, and subjective tolerability [4]. Obesity, cardiovascular disease, and metabolic syndrome are also factors that impact a patient's life prognosis and QOL [5, 6].

Cook et al. [7] reported that for stable outpatients with schizophrenia in a real-world setting, switching to an atypical antipsychotic can result in sustained, significant improvement in clinical response and QOL, as well as in reduced need for hospitalization and community support. Chaudhry et al. [8] indicated that psychiatrists consider switching antipsychotic therapy if excessive sedation, extrapyramidal symptoms, unacceptable weight gain, hyperglycaemia, or dyslipidaemia occur. In general, switching is more likely to be considered for symptomatic adverse events than for laboratory abnormalities.

Aripiprazole plays an inhibitory role as a D2 receptor antagonist when dopamine is released excessively in the brain. On the other hand, it acts as a dopamine agonist when dopamine is insufficient. It is called a dopamine D2 receptor partial agonist [9] and has the partial agonistic effects of a dopamine D2 receptor and a serotonin 5-HT1A receptor and the antagonistic effect of a serotonin 5-HT2A receptor, while it has a minimal anticholinergic or antihistaminergic effects, which are related to side effects. Consequently, aripiprazole has fewer metabolic and cholinergic side effects, when compared with other antipsychotic drugs [10, 11].

Abnormal metabolism may increase the risk of coronary heart disease and diabetes [12], however, aripiprazole is considered to be less likely to cause weight gain and abnormal glucose and lipid metabolism [13, 14]. Thus, it is possible that treatment with aripiprazole can prevent the onset of coronary heart disease and diabetes, compared with treatment with other drugs [15]. Also, it is reported that switching to aripiprazole could lead to a significant decline of prolactin and improvement in sexual dysfunction, resulting in better patient compliance [16]. Aripiprazole is an atypical antipsychotic associated with a reduction in prolactin level in antipsychotic-induced hyperprolactinemia [17]. Furthermore, compared with olanzapine, quetiapine, or risperidone, aripiprazole is reported to have better effects on the self-care ability and the QOL of patients [18]. Additionally, fewer side effects generally lower the cost of treatment as well as increasing patient's long-term health benefits. 

The purpose of this retrospective study was to evaluate changes in clinical indicators which influence QOL before and after switching antipsychotic medication to aripiprazole.

## 2. Methods

In this retrospective chart review, the subjects whose antipsychotic was switched to aripiprazole were evaluated. Subsequently, the effects of the switching and quality of life were considered. The subjects were then divided into two groups responsive and nonresponsive. 

Changes in the following clinical indicators before and after the switching were analyzed: (1) daily dosage of antipsychotics and antiparkinsonian drugs, (2) the Brief Psychiatric Rating Scale (BPRS), (3) body mass index (BMI), (4) glucose and lipid metabolism, (5) bowel movement pattern, (6) falling risk score, (7) degree of psychiatric nursing needs, and (8) activities of daily living (ADL's). 

The setting for this study was a long-term care unit in a psychiatric hospital in Japan with approximately 500 beds. Approval for the research study was obtained from the Ethical Board of Mifune Hospital. The sample consisted of 27 charts of chronic, long-term hospitalized subjects diagnosed with schizophrenia who had been switched to aripiprazole during the research period of January 2006 to March 2010. The sample consisted of 17 men and 10 women, ages 34–82.

For each of the clinical indicators, the Wilcoxon signed-rank test was used to examine whether or not significant differences occurred after the switching were examined by using. Then, cross-tabulation and the Fisher's exact test were conducted. Data analysis was performed using SPSS11.0J for Windows. 

The following eight clinical indicators were used to measure the responsiveness of subjects to aripiprazole. Each clinical indicator was reviewed prior to the switch to aripiprazole, two months after the switch, and when the aripiprazole dose had become constant. Clinical indicator measurements were defined as follows.

1. Daily dose of antipsychotic and antiparkinsonian medication: potency equivalents for antipsychotic drugs are required to guide clinical dosing and for designing and interpreting research studies. The daily dosage of antipsychotic drugs was converted to a chlorpromazine (CP) equivalent, and the daily dosage of antiparkinson drugs was converted to a biperiden (BP) equivalent [19].
2. BPRS: the Japanese version of the BPRS is one of the most frequently used instruments for evaluating psychopathology in patients with schizophrenia. Each symptom is rated 1–7 and depending on the version, a total of 18 items are scored.
3. BMI: (20–24 kg/m^2^). the values in brackets represent the normal range of each item.
4. Glucose and lipid metabolism: the following measurements were evaluated: total protein (TP: 6.4–8.2 g/dL), albumin (ALB: 3.7–5.2 g/dL), albumin-globulin ratio (A/G ratio: 1.1–2.0), total cholesterol (T-cho: 130–240 mg/dL), hemoglobin (Hb: male 13.5–17 g/dL; female 11.5–15 g/dL), and preprandial blood glucose level (60–110 mg/dL).
5. Bowel movement patterns: bowel movements were categorized as “one bowel movement per day,” “one bowel movement in two days,” “one bowel movement in three days,” “one bowel movement in four days,” and “one bowel movement in five days.”
6. Falling risk score: the falling risk score consists of 3 levels: Risk level 1: possibility of falling (score 1–5); Risk level II: likelihood of falling (score of 6–15); Risk level III: frequently falling (score of 16 or more).

The falling risk score was determined by rating the following 6 items.

 
  - Age: 60 y/o and greater;
  - hospitalization history: history of falling or fainting during prior hospitalizations;
  - functional disabilities: deterioration of the feet and waist, loss in muscle strength, use of a wheelchair, a stick, or a walker, the need of support for movement, experiencing a wobble or not, and the insertion of any route and/or drains in the body;
  - recognition abilities: disorientation, clouding consciousness, confusion, dementia, abilities to judge and understand, restless behavior, and the difficulty in relearning due to memory decline;
  - medicine: analgesic drugs, narcotic drugs, sleeping tranquilizers, antiparkinsonian drugs, antihypertensive diuretics, enema laxative, and chemotherapy;
  - egestion: urinary or fecal incontinence, frequent micturition, the need of egestion during sleeping, and the distance to the toilet.
(7) Degree of psychiatric nursing care: the degree of the need of nursing followup was evaluated by using the following levels: (a: followup is needed all the time); (b: constant followup is needed); and (c: continuous followup is not necessary, or followup after a longer interval than B).
(8) Activity of daily living (ADL): life was evaluated using the following definitions: (I: not being able to do any things by him/herself); (II: being able to do some things by him/herself, but having many things s/he cannot do); (III: being able to do most things, but having a problem in his/her independent activities); (IV: being able to do many things independently, but having a problem in social adaptation).

## 3. Results

### 3.1. Demographics

The total subjects of this research were 17 men and 10 women. Fourteen subjects (10 men and 4 women) were responsive, and 13 subjects (7 men and 6 women) were nonresponsive. The average age for the responsive group was 66.7 ± 9.8 years old (52–82 years old) and in the nonresponsive group, the average age was 54.5 ± 12.1 years old (34–70 years old). Results revealed that 14 subjects positively responded to the switch to aripiprazole while 13 subjects either had no change or negative responses to the switch to aripiprazole. The group of the subjects who were changed to aripiprazole without worsening psychiatric conditions was defined as the responsive group. The group of subjects who were changed to other antipsychotics due to worsening psychiatric conditions after switching to aripiprazole was defined as the nonresponsive group. 

### 3.2. The Daily Dosage of Antipsychotics and Antiparkinsonian Drugs

The average period of time from the start of switching to switching completion (or discontinuation of switching) was 17.0 ± 10.0 months in the responsive group and 12.5 ± 11.5 months in the nonresponsive group. By the end of switching, 5 subjects in the nonresponsive group switched to olanzapine, 3 switched to risperidone, 2 switched to quetiapine, one switched to levomepromazine, one switched to blonanserin, and one used bromperidol in combination with blonanserin.

There was no significant difference in the dosage of antipsychotics (CP equivalent) and antiparkinsonian drugs (BP equivalent) in the two groups before the switching. However, in the nonresponsive group, the daily dosage of antipsychotics tended to be higher after switching (*P* = 0.08). Also, although there was no significant difference in the daily dosage of antiparkinsonian drugs before and after the switching in either of the groups, the responsive group showed a tendency to lower dosage after the switching (*P* = 0.07) (Table 1).

Table 2 shows the result of daily changes of antipsychotics by using cross-tabulation. For 71% of the subjects in the responsive group and 23% in the nonresponsive group, the dosage of antipsychotics was decreased after the switching. The number of subjects whose dosage of antipsychotics was decreased was significantly larger in the responsive group than in the nonresponsive group (*P* = 0.03).

### 3.3. Psychiatric Condition and Glucose/Lipid Metabolism

The BPRS values were significantly decreased after the switching in the responsive group (*P* = 0.04), but there was no change in the nonresponsive group. There was no change in TP, ALB, A/G ratio, and preprandial blood glucose level after the switching in both of the groups. While there was a tendency toward a decline in the BMI after the switching in the responsive group (*P* = 0.06), no significant change was observed in the nonresponsive group. In the nonresponsive group, T-cho increased significantly (*P* = 0.01), whereas no significant change was observed in the responsive group (Table 1).

### 3.4. Clinical Evaluation by Nursing Observation

No change was observed in bowel movement frequency, the scores for falling risk, or the ADL scores before and after the switching in either of the groups. In addition, regarding the degree of psychiatric nursing needs, there was no significant difference observed (Table 3).

## 4. Discussion

### 4.1. The Effects of Switching to Aripiprazole

By switching the antipsychotics to aripiprazole, there were some significant improvements in psychiatric condition in the responsive group, showing the effectiveness of switching to aripiprazole. As the dosage of antiparkinsonian drugs in the responsive group tended to decline after the switching, it is suggested that extrapyramidal side effects were reduced by switching to aripiprazole [20]. This was considered to be because of the pharmacological action of aripiprazole acting as a D2 receptor partial agonist. Also, as antiparkinsonian drugs are known to cause anticholinergic side effects and cognitive dysfunction, the discontinuance of, or decrease in the dosage of antiparkinsonian drugs is effective in long-term treatment of schizophrenia. 

On the other hand, there was no significant change in the dosage of antipsychotics in the nonresponsive group who were switched to other antipsychotics. However a tendency toward a higher dose of antipsychotics after the switching may indicate a temporarily worsening psychosis. 

A significant increase in lipid levels was observed after the switching in the nonresponsive group. Eight of 13 subjects in the nonresponsive group were switched to other drugs, such as olanzapine and quetiapine, which were likely to affect glucose and lipid metabolic abnormality.

It is reported that aripiprazole is less likely to cause various side effects including glucose and lipid metabolic abnormality [21]. In this research, the glucose and lipid levels in the responsive group who switched to aripiprazole were not significantly changed after the switching, but the BMI indicated a tendency to decline in the responsive group. This suggests that switching to aripiprazole returned subjects to their prior body weight after prior weight gain.

According to Kolotkin's research [22], persons taking aripiprazole experienced decreased weight and improved weight-related QOL compared with other antipsychotics. The prevalence of metabolic syndrome is increased 2-3-fold in schizophrenic patients with serious mental illness. Metabolic syndrome and other cardiovascular risk factors are highly prevalent in people with schizophrenia [23], leading to a life expectancy approximately 20% shorter than that of the general population [24, 25]. Consequently, reducing the incidence of glucose- and lipid-related side effects could lead to reduction in use of other drugs to counter these side effects and a huge reduction in the risk of developing other diseases. Studies by Croxtall [26] and Ganguli et al. [27] support this study's results indicating that aripiprazole as having a low risk of metabolic syndrome.

### 4.2. The Improvement of QOL Related to Switching to Aripiprazole

Nursing evaluations indicated the bowel movement frequency, falling risk scores, the degree of psychiatric nursing needed, and the ADL score remained unchanged. This might be because the assessment was carried out only two months after switching from the previous medications; thus, it will be necessary to observe this factor in the long term. However, there was a tendency toward increased proportion and degree of psychiatric nursing needs in the responsive group. The increase in proportion of continuous followup despite no change in the life dependence levels suggests the effectiveness of the switch to aripiprazole, indicating that patients with aripiprazole could live independently. 

Before the switching, 4 subjects in the responsive group were in the category, “continuous followup is needed all the time,” but this declined to one subject after the switching. This suggests that the patients needed less nursing assistance. This result suggests that switching to aripiprazole might contribute to reducing the burden on nurse as well as to the transition of patients to community living status.

It is reported that patients with schizophrenia who have been chronically treated with high-dose antipsychotic polypharmacy are more dependent on psychiatrists and nurses [28]. The minimal effective dose of antipsychotics can reduce the risk of side effects and lead to greater self-managing and self-care. This not only reduces the burden for caregivers in drug administration for patients, but also improves patients' drug adherence. 

To improve quality of life and decrease metabolic syndrome in patients with schizophrenia, it is essential for caregivers to support patients' drug administration with a minimal effective dose, and aripiprazole is considered to be one of the most effective options available. In order to make the treatment with aripiprazole more effective, physical exercise and nutritional instruction based on the evaluation of glucose and lipid metabolism are also necessary. Also, for patients to move toward a healthy lifestyle, a comprehensive support system is required. Such a system should include collaboration between the patient's various specialists and should take into account the patient's level of subjective side effects and discomfort in daily life.

The limitations of the present study include the small sample size and the retrospective design using secondary data. Also, the different switching methods might affect the degree of switching success, and switching protocols were dependent on several physicians, which might also affect outcomes.

## 5. Conclusions

The results of this research suggest that switching to aripiprazole improves psychiatric symptoms and leads to fewer side effects for persons diagnosed with schizophrenia. This could improve a person's quality of life, medication adherence, and perhaps lead to living in the community. It is important to improve patients' medication adherence through patient education of side effects and drug advantages in order to make full use of aripiprazole. In addition to drug administration, it is also important that doctors, pharmacists, nutritionists, and nurses collaborate to support patients' movement toward a healthy lifestyle.

## Figures and Tables

**Table 1:** Changes of clinical indicators of before and after switch to aripiprazole.

	Responsive group (*n* = 14)				Nonresponsive group (*n* = 13)			
	Before (Mean ± SD)	After (Mean ± SD)	*Z* value	*P* value*	Before (Mean ± SD)	After (Mean ± SD)	*Z* value	*P* value*
Mean daily dosage								
Antipsychotics (CP equivalent; mg)	489.4 ± 335.5	448.6 ± 184.3	−0.28	0.78	527.8 ± 359.9	771.0 ± 514.5	−1.73	0.08
Antiparkinson drug (BP equivalent; mg)	1.0 ± 1.5	0.23 ± 0.6	−1.80	0.07	1.5 ± 2.4	0.9 ± 1.8	−0.94	0.35
Psychosis								
BPRS total score	32.6 ± 8.9	28.1 ± 12.2	−2.28	0.04	33.7 ± 10.2	32.8 ± 10.9	−0.57	0.58
Glucose and lipid metabolism								
TP (g/dL)	6.7 ± 0.5	6.9 ± 0.9	−1.20	0.23	6.5 ± 0.7	6.7 ± 0.6	−0.79	0.43
ALB (g/dL)	4.0 ± 0.4	3.9 ± 0.7	−1.16	0.25	3.9 ± 0.6	4.0 ± 0.4	−0.05	0.96
A/G ratio	1.4 ± 0.3	1.4 ± 0.3	−0.56	0.58	1.6 ± 0.3	1.5 ± 0.3	−1.30	0.20
T-cho (mg/dL)	172.9 ± 36.8	178.5 ± 41.8	−0.80	0.42	166.2 ± 36.5	184.5 ± 36.0	−2.67	0.01
Hb (g/dL)	11.4 ± 2.4	12.1 ± 2.3	−1.04	0.30	11.3 ± 2.7	12.1 ± 3.0	−0.94	0.35
Preprandial blood glucose level (mg/dL)	90.4 ± 17.1	86.1 ± 14.0	−0.73	0.46	79.0 ± 26.8	80.7 ± 7.1	−0.45	0.66
BMI	21.8 ± 3.8	20.8 ± 3.6	−1.85	0.06	22.7 ± 6.0	21.9 ± 5.9	−1.10	0.27

CP: chlorpromazine, BP: biperiden, BPRS: brief psychiatric rating scale, TP: total protein, ALB: albumin, A/G ratio: albumin-globulin ratio, T-cho: total cholesterol, Hb: hemoglobin, and BMI: body mass index. *two-tailed asymptotic significance probability (Wilcoxon signed-rank test).

**Table 2:** Changes in dosage of antipsychotics per day.

	Responsive group (*n* = 14)		Nonresponsive group (*n* = 13)		*χ* ^ 2^ value	*P* value*
	Number of subjects	%	Number of subjects	%
Decreased	10	71	3	23	7.08	0.03
Increased	4	29	8	62		
No change	0	0	2	15		

*Two-tailed asymptotic significance probability (Fisher's exact test).

**Table 3:** Changes in clinical evaluation by observation of nursing.

		Responsive group (*n* = 14)						Nonresponsive group (*n* = 13)					
		Before		After				Before		After		*χ* ^ 2^ value	*P* value*
		Number of subjects	%	Number of subjects	%	*χ* ^ 2^ value	*P* value*	Number of subjects	%	Number of subjects	%
The bowel movement	One bowel movement per day	7	50	5	35.7	0.88	0.20	4	30.8	5	38.5	2.60	0.15
	One bowel movement in two days	6	42.9	8	57.1			9	69.2	6	46.2		
	One bowel movement in three days	1	7.1	1	7.1			0	0	1	7.7		
	One bowel movement in four days	0	0	0	0			0	0	0	0		
	One bowel movement in five days	0	0	0	0			0	0	1	7.7		

Falling risk	Risk level I: possibility of falling	9	64.3	7	50	0.58	0.45	5	38.5	7	53.8	2.03	0.36
	Risk level II: likelihood of falling	5	36	7	50			8	61.5	5	38.5		
	Risk level III: frequently falling	0	0	0	0			0	0	1	7.7		

Degree of psychiatric nursing needs	a: followup is needed all the time	4	28.6	1	7.1	2.19	0.14	6	46.2	3	23.1	1.56	0.10
	b: constant followup is needed	0	0	0	0			3	23.1	4	30.8		
	c: continuous followup is not necessary	10	71.4	13	92.9			4	30.8	6	46.2		

ADL	I: not being able to do things by him/herself	1	7.1	0	0			1	7.7	2	15.4		
	II: being able to do some things by him/herself, but having many things s/he cannot do	2	14.3	5	35.7	5.41	0.17	7	53.8	5	38.5	1.22	0.17
	III: being able to do most things, but having a problem in his/her independent activities	10	71.4	5	35.7			4	30.8	4	30.8		
	IV: being able to do quite many things independently, but having a problem in social adaptation	1	7.1	4	28.6			1	7.7	2	15.4		

ADL: activity of daily living, *asymptotic significance probability (Fisher's exact test).
